# Defining Established and Emerging Microbial Risks in the Aquatic Environment: Current Knowledge, Implications, and Outlooks

**DOI:** 10.1155/2011/462832

**Published:** 2010-09-27

**Authors:** Neil J. Rowan

**Affiliations:** Department of Nursing and Health Science, School of Science, Athlone Institute of Technology, Dublin Road, Athlone, Co. Westmeath, Ireland

## Abstract

This timely review primarily addresses important but presently undefined microbial risks to public health and to the natural environment. It specifically focuses on current knowledge, future outlooks and offers some potential alleviation strategies that may reduce or eliminate the risk of problematic microbes in their viable but nonculturable (VBNC) state and *Cryptosporidium* oocysts in the aquatic environment. As emphasis is placed on water quality, particularly surrounding efficacy of decontamination at the wastewater treatment plant level, this review also touches upon other related emerging issues, namely, the fate and potential ecotoxicological impact of untreated antibiotics and other pharmaceutically active compounds in water. Deciphering best published data has elucidated gaps between science and policy that will help stakeholders work towards the European Union's Water Framework Directive (2000/60/EC), which provides an ambitious legislative framework for water quality improvements within its region and seeks to restore all water bodies to “good ecological status” by 2015. Future effective risk-based assessment and management, post definition of the plethora of dynamic inter-related factors governing the occurrence, persistence and/or control of these presently undefined hazards in water will also demand exploiting and harnessing tangential advances in allied disciplines such as mathematical and computer modeling that will permit efficient data generation and transparent reporting to be undertaken by well-balanced consortia of stakeholders.

## 1. Viable But Nonculturable Forms of Waterborne Bacteria

### 1.1. Background

Since the introduction of the concept or sublethally injured or viable but nonculturable (VBNC) cells by Byrd and Colwell in the 1980's [1], there is increasing evidence for the existence of such a state in microbes, particularly in the aquatic environment that elicits a myriad of interrelated sub-lethal microbial stresses such as nutrient starvation and osmotic stress [2, 3] (Table 1). This is a cause for concern because of evidence that microbial pathogens in such a state may still retain their capacity to cause infections after ingestion by fish, animals, or by humans, despite their inability to grow under conditions employed in laboratory-based procedures for determining their presence in water [4]. Albeit currently unknown in terms of its severity or scope, it is now generally appreciated that heavily stressed pathogenic microbial species existing in a VBNC (or not immediately culturable state) may potentially pose as yet an undefined risk to public health, which is attested by the fact that there is increasing evidence to support the viewpoint that stressed cells in this quiescent state may actually be more virulent than well-fed laboratory-tamed microorganisms due to augmented virulence factor expression. Xu et al. [5] were the first to bring experimental evidence of the existence of VBNC state in pathogenic bacteria, where they showed that *E. coli* and *V. cholera* cells that were suspended in artificial seawater quickly lost their ability to grow on the culture media normally used for their detection. 

### 1.2. Definition

According to Oliver [16], a bacterium in the VBNC state is defined as “a cell which is metabolically active, which being incapable of undergoing the cellular division required for growth in or on a medium normally supporting grown of that cell.” Besnard et al. [17] suggest that the transition to the VBNC state in *L. monocytogenes* represents a survival strategy that bacteria can adopt under adverse conditions (starvation, salt stress, etc.). VBNC microorganisms are considered to represent a subpopulation of cells that are unable to grow in the usual culture media and cannot resuscitate by traditional resuscitation techniques, but yet remain physically active for several functions such as cellular elongation [18], respiratory chain activity [6, 7, 17], or incorporation of radio-labelled substrates [8]. For example, Cappelier and coworkers [4] recently reported that avirulent VBNC cells of *L. monocytogenes* incubated in filtered sterilized distilled water need the presence of an embryo to be recovered in egg yolk and regain virulence after recovery. The VBNC state was observed after a 25 to 47 days incubation period (concentration of culturable cells less than 1 colony forming unit per mL). 

### 1.3. VBNC State and Occurrence of Atypical Morphological Types

As microorganisms are extremely diverse and dynamic, it is not surprising that the many different types of microbial species present in the water environment exist in a number of physiological states that possess different requirements for survival and to sustain growth. Indeed, the number of waterborne bacteria in which the VBNC state has been reported has greatly increased, particularly in recent times that reflect technological advances. For instance, *Campylobacter jejuni* has been reported to exist in two different cellular morphotypes, where the atypical coccus-form (currently associated with nongrowing VBNC state) occurs in water under extended nutrient depletion conditions [19]. A number of different research groups have reported that these atypical culture forms are still capable of infection mice and poultry [20]. Moreover, Rowan and coworkers [21] recently reported on that different culture morphotypes of *L. monocytogenes* generated in water after exposure to novel pulsed-plasma gas-discharge treatment can survive internalization by human polymorphonuclear leukocytes. While Lindbäck et al. [9] reported that the ability to enter into an avirulent VBNC form is widespread among *L. monocytogenes* isolated from salmon, patients and the environment. *L. monocytogenes* were tested for virulence in a cell plaque assay and by intraperitoneally inoculation in immunodeficient RAG1 mice. Moreover, Moreno et al. [22] described successions in cellular alterations in *Helicobacter pylori* NCTC 11637 after inoculation into chlorinated drinking water. They concluded that *H. pylori* could survive disinfection practices normally used in drinking water treatment in the VBNC form, which would allow them to reach final consumption points and, at the same time, enable them to be undetectable by culture methods. Whereas Kastberg et al. [23] recently reported that *L. monocytogenes* cells, whether planktonic or attached, were homogenous with respect to sensitivity to acidic disinfectants at the single-cell level.

### 1.4. Use of Fluorescent Redox and Other Enzymatic Probes

Directvisualization of actively respiring bacteria is gaining in popularity amongst research groups investigating this VBNC state [3, 6, 7]. Researchers have exploited use of metabolic staining to reveal an underestimation in the level of microbial survival compared to similar samples cultured on traditional agar plates. Recent research in our laboratory has also shown that subpopulations of waterborne pathogens such as *E. coli* and *Pseudomonas* spp. treated with novel pulsed-power disinfection technologies (such as use of pulsed-plasma gas-discharge technology that will be expanded on later in this paper) were capable of reducing the redox dye 5-cyano-2, 3-ditolyl tetrazolium chloride (CTC) that is an indicator of electron acceptor function, yet similarly treated samples were unable to form colonies on a variety of laboratory-based culture media [6, 7]. This corroborates more recent research undertaken by Sawaya and coworkers [3] who used a combined 4'6-diamidion-2-phenylindole (DAPI) and CTC stains to highlight the occurrence of physiologically active bacteria in river and wastewater treatment plants that were much higher than those obtained by plate counting. These researchers also reported that microscopic viable bacteria were more chlorine resistant than culturable bacteria. That said, a demonstration of active respiration does not necessarily infer that these stressed bacteria are capable of future growth. As numerous researchers continue to report on the use of redox stains for highlighting differences in agar-plate counts, it is important that we holistically explore and identify specific microbial cues at the cellular level which govern the transition to the VBNC state along with exploiting commensurate advances in media formulations that are tailored for optimal resuscitation of these sublethally stressed cells (cited in [10, 24]). The latter authors showed that the addition of a commercially available antioxidant Oxyrase and a heat-stable autoinducer of growth secreted by enterobacterial species in response to norepinephrine, resuscitated *E. coli*, and *Salmonella enteric* serovar Typhimurium that were stressed by prolonged incubation in water microcosms.

### 1.5. VBNC State and Cell Suicide Phenomenon

 Advancing the earlier pioneering work of Dodd et al. [25] and Aldsworth et al. [26], who previously postulated that self-destruction or “cell suicide” may be attributed to sublethally stressed or damage microbes being incapable of coping with oxidative burst when rapidly growing on nutrient rich media, it is important that we also exploit advances in toxicology (such as methods exploring cellular apoptosis and necrosis, cell membrane lipid peroxidation assays, and nuclear chromatin/comet assays) that will provide valuable insights into the possible role of intracellular free radicals in combination with the direct physical action of the applied environmental stress on the generation and persistence of VBNC organisms in water. Indeed, Servais and coworkers [12] recently reported that certain environmental factors such as nutrient scarcity and solar irradiation lead to a high proportion of VBNC *E. coli* in freshwater. The latter fecal microorganisms are brought into freshwater environments mainly through wastewater release, surface runoff, and soil leaching. Interestingly, modified guidelines for bathing water states that enumeration of *E. coli* will replace total coliforms and fecal (also called thermotolerant) coliforms as bacterial indicators of water quality [27]. The latter authors articulated that the number of *E. coli* in freshwater is systematically underestimated by traditional culture-based methods such as multiple tube fermentation and or membrane filtration techniques), which is cause for concern from a public health perspective. On a related theme, numerous researchers have also recently reported on the occurrence of “bacterial autophagy” (i.e., microbial adaptation to autophagic microbicidal host immune cell defences), which is an important cell survival process initiated during nitrogen starvation conditions [28].

### 1.6. Molecular-Based Techniques for Rapid Detection and Classification of VBNC Organisms

Among the minority of bacteria that have been discovered it is estimated that more than 90% are as yet nonculturable as attested by the fact that International Committee on Systematic Bacteriology has recognised a new category for nonculturable bacteria that it named “*Candidatus*” for which phylogenetic relatedness has been determined by amplification and sequence analysis of prokaryotic RNA genes with universal prokaryotic primers and authenticity has been verified by use of *in situ* probes. Such nonculturable organisms may only be detected by use of such molecular techniques based on probes such as 16S and 23S rRNAs or on determination of mRNA, either by quantitative real-time PCR and/or by fluorescent techniques such as *in situ* hybridization (FISH), microradiography, epifluorescence microscopy, and flow cytometry [2, 11, 12]. While Fiksdal and Tryland [13] advocated use of rapid enzyme assays for monitoring of water quality, which may also detect organisms in the injured or VBNC state. Garcia-Armisen and Servais [11] showed that the ratio of direct viable- (DVC-) FISH count and the culturable count increased with decreasing abundance of culturable *E. coli* in river water, and therefore the slope of the linear-log-log correlation of DVC-FISH versus colony forming unit numbers was less than one. The authors hypothesized that the more stressful conditions, such as nutrient deprivation and increase solar stress at low turbidities met in low contaminated environments, were responsible for the larger fraction of VBNC *E.coli*. As mentioned previously by many research groups [13, 29–33], all field trials plotting log *β*-D-galactosidase (GALase) activity or log *β*-D-glucuronidase (GLUase) activity versus log culturable target bacteria in fresh or marine water have shown regression straight lines with a slope less than one, suggesting that enzyme activity calculated per culturable indicator bacteria increases when their numbers decrease (e.g., when sewage effluent becomes diluted in receiving waters). Pure culture studies of *E. coli* have also shown that after exposure to other types of extrinsic stress such as chlorination, the GALase activity is less reduced than the direct viable count [29]. While Zimmerman and coworkers [34] demonstrated that *E. coli* can be present in higher numbers in recreational water samples using fluorescent antibody direct viable counting that what are detected with standard culture methods. The latter advances the early landmark study of Bjergbaek and Roslev [14] who reported on the occurrence and persistence of VBNC *E. coli* in nondisinfected drinking water using different cultivation dependent methods, fluorescence in situ hybridization (FISH) using specific olignucleotide probes, direct viable counts (DVC), and by enumeration of GFP-tagged *E. coli* (green fluorescent protein, GFP). These studies specifically reemphasises the need for a rapid, accurate, and precise method for detecting health risks to humans from contaminated water. 

Other likely candidate methods for efficient and rapid-detection of VBNC bacteria in the aquatic and soil environments include RNA-based genotypic approaches. Dunaev et al. [2] recently reported on the rapid and accurate quantification of VBNC pathogens in biosolids via monitoring and quantifying stress-related genes in *Salmonella* spp. using cDNA microarrays combined with quantitative reverse transcription polymerase chain reaction (qRT-PCR). Quantification of mRNA was correlated to cell viability and their ability to grow. Okabe and Shimazu [35] also describe detection of host-specific *Bacteroides-Prevotella* 16S rRNA genetic markers (total, human, cow- and pig-specific) as promising alternative indicators for identifying the sources of fecal pollution in environmental water because of their abundance in the feces of warm-blooded animals. The authors clearly state that detection of aforementioned genetic markers mainly reflected the presence of VBNC *Bacteroides-Prevotella* cells in water, suggesting that seasonal and geographical variations in persistence of these host-specific *Bacteroiides-Prevotella* 16S rRNA genetic markers must be considered if used as alternative fecal indicators in environmental waters. This corroborates emerging studies that suggest that increases in coliform concentration after STP and dewatering processes may be attributed to cells going into VBNC state implying traditional coliform enumeration methods are not sufficient to determine number of viable cells. This also reinforces common viewpoint shared by many scientists that a variety of problematic bacteria can enter VBNC states as a survival response when exposed to deleterious environmental stresses such as nutrient starvation, osmotic stress, and so forth. However, in order to gain a greater appreciation of environmental and public health risks associated with persistence of microbial pathogens in different culturable states, it is imperative that we acquire an understanding of the interrelated molecular responses governing tolerance to these applied sub-lethal stressors. Such as detailed investigations reported by Brackman et al. [15] who recently found that autoinducer- (AI-) 2 quorum-sensing inhibitors affected starvation response and reduce virulence in several *Vibrio* species, most likely by interfering the signal transduction pathway at the level of LuxPQ.

### 1.7. Risk Assessment and Predictive Modeling for Management of VBNC State Organisms in Water

The aforementioned detection methods are not as yet commonly used for routine measurement as they either are not simple enough and/or the equipment is too expensive. Therefore, in terms of enabling effective risk-based assessment and environmental management to address the occurrence and persistence of VBNC pathogens in water, critical data must be acquired to convert VBNC-phase potential pathogens from unknown to known and defined hazards. In terms of satisfying these information gaps, accurate enumeration of total microbial load and real time identification to the various types present in water are pivotal to hazard identification and characterization, which will contribute significantly to assuring water safety. Regarding the latter, Smeets et al. [36] recently described improved methods for modelling drinking water safety using a robust quantitative microbial risk assessment (QMRA), where a case study monitored *Campylobacter* data for rapid sand filtration and ozonation processes. This study showed that currently applied methods do not predict monitored data used for validation. Therefore, underestimation in the levels of problematic microbes in water, and failure to identify the presence of pathogenic organisms in representative water samples also pose significant threats to public health. 

### 1.8. Use of Advanced Detection Techniques to Investigate Virulence Potential in VBNC State Organisms

Greater information is also required on elucidating the existence of commonly shared cellular mechanisms (and associated gene expression regulators and gene markers) that govern cellular conversion to this VBNC state. Moreover, there is a dearth of knowledge regarding specific underlying molecular and associated cellular mechanisms governing transition and persistence of waterborne microorganisms in this VBNC state, in addition to obviously establishing what specific environmental conditions or triggers cause these changes in culturable state. Greater studies are also required to investigate the presence and role of this VBNC state in microbial pathogens that cause disease in the aquatic natural setting such as in fish.Taking for example,*Flavobacterium psychrophilum*, the causative agent of rainbow trout fly syndrome and cold water disease in salmonids, where subpopulations were still culturable after starvation for 300 days in sterilised fresh water. The virulence of starved *F. psychrophilum* was maintained for at least 7 days after the transfer of the bacterial cells to fresh water [37]. The VNBC state was earlier reported for this fish pathogen by Madetoja and Wiklund [38] who revealed differences between enumeration using advanced immunoflourescence and genetic probes (i.e., nested PCR) compared to that of using traditional agar plate cultivation. While the long-term survival cellular responses to salinity, temperature and starvation have been studies in the eel pathogen *Vibrio vulnificus* for over a decade [39]. To combat such severe microbial infections affecting fish, many researchers have advocated water recirculation and good management as potential methods to avoid disease outbreaks particularly with *F. psychrophilum* [40]. Thus, highlighting the importance of augmenting water quality through improvements in wastewater treatment, which should be augmented in efficiency and capacity to cater for under-appreciated and for emerging microbial threats.

### 1.9. Addressing Scientific Shortfalls in VBNC to Inform Policy

To comprehensively address such issues, detailed analysis of the proteome that arise during this morphological transition will enable identification and characterization of key proteins that are responsible for this VBNC state. In addition, tandem use of microarray gene analysis along with real-time quantitative PCR will help unravel specific gene functions and will pin-point over-arching regulatory framework(s). The aforementioned studies should also be carried out in parallel with developing improved protocols for resuscitating VBNC organisms, particularly availing of advances made in fermentation technology such as exploiting chemostat-based bioreactor approaches that can simulate and monitor multiple environmental stresses (either applied simultaneously or sequentially) over extended time periods. Indeed, Kooi and coworkers [41] recently exploited use of a chemostat to report on the dynamic behaviour of simple aquatic ecosystems with emphasis on nutrient recycling in order to monitor toxicants. Thus, use of the latter provides a useful vehicle for investigating the effects of deleterious microbial stressors on the structure and functioning of ecosystems. Other related studies that merit attention include the development of more rapid, user-friendly, field-based technologies that will reliably and repeatably detect and quantify various waterborne pathogens that may persist in different culture states. Less than four hours was recommended as “rapid” by Noble and Weisberg [42] in a recent review of rapid detection methods for bacteria in recreational waters. Due to plethora of different detection methods currently being developed combined with obvious interlaboratory variability in terms of associated operating protocols, it is imperative that we develop and agree upon a standardized battery of reliable tools for detecting and quantifying culturable and nonculturable bacteria so as to establish future unified, quantitative-risk management protocols for monitoring our aquatic environments. The latter will greatly facilitate our endeavours to collectively comply with emerging environmental policies such as EU's Water Framework Directive.

## 2. The Waterborne Enteroparasite: Cryptosporidium

### 2.1. Background

*Cryptosporidium species* have emerged over the past decades as major waterborne pathogens causing gastroenteritis in humans [43]. The occurrence of the environmentally resistant thick-walled oocyst stage of this organism has become a worldwide concern due to its resistance to disinfection with chlorine at concentrations typically applied in drinking water treatment plants (2 to 6 mg/L) [44]. Thus, the control of *Cryptosporidium* oocysts remains a major challenge for drinking water utilities due to fact that the common chemical disinfectants-free and combined chlorine, when used singly, are practically ineffective for inactivating this protozoan under conditions encountered in most treatment facilities [45].* Cryptosporidium *spp. are found ubiquitously and the transmission “oocyst-stage” may remain viable for several months in the aquatic environment. This protozoan is transmitted via the faecal-oral route, where consumption of contaminated drinking water and use of recreational water are major sources of infection [46]. *Cryptosporidium *oocysts are resistant to conventional disinfectants at concentrations and exposure times commonly used, and their infectious doses in humans have been estimated to be as low as 30 oocysts [47]. Indeed, more than 160 waterborne outbreaks of cryptosporidiosis have been reported globally, with the greatest documentation occurring in the US and the UK [46, 48]. 

This situation has become a major concern for water authorities and consequently, significant modifications to drinking water regulations have been proposed for the detection and surveillance of this protozoan in the US and in other developed countries [49]. The new European Drinking Water Directive advocates that all the state members should provide drinking water supplies with absence of pathogenic organisms [50]. However problems associated with the determination of oocysts viability/infectivity make the establishment of maximum acceptable concentrations very difficult, and concentrations of ≥3–30 oocysts/100 litres in treated water have been proposed as action levels [51]. Recent contamination of drinking water supplies in the west of Ireland have led to a significant number of confirmed cases of cryptosporidiosis, intimating that the use of conventional decontamination methods failed to eliminate this enteroparasite in treated water (cited in Garvey et al. [52]). This situation has become a major concern for water authorities and consequently, significant modifications to drinking water regulations have been proposed for the detection and surveillance of this protozoan in the US and in other developed countries [50].

### 2.2. Alternative or Complementary Decontamination Methods

Development of alternative methods of *Cryptosporidium *disinfection for water applications (such as ozone and/or UV) has been hindered by the uncertainty surrounding efficacy of using *in vitro* surrogate viability assays due to their overestimation of oocysts survivors posttreatments and the lack of critical data on the preferred use of *in vitro* cell culture and/or *in vivo* animal-based infectivity assays to determine interrelated factors governing repeatable disinfection of oocysts suspended in water [53]. Although recent studies that utilised at least 20 different cell lines have advocated the preferential use of the human ileocecal adenocarcinoma HCT-8 cell line as an equivalent *in vitro* method to that of using the “gold standard” mouse assay for measuring infectivity of *Cryptosporidium* [44], there has been limited evidence to date on the combined use of these approaches for assessing critical operational parameters governing pulsed UV light (PUV) as a means of disinfecting water contaminated with this enteroparasite [53]. This study demonstrated that there is good agreement between use of *in vitro* culture-*q*PCR and the SCID-mouse-infectivity assays for evaluating the disinfection efficacy of pulsed UV for inactivating *C. parvum* suspended in saline, thus reducing the requirement to unnecessarily use animals for these particular research studies. However, the authors recommended that pressing investigations are needed to comprehensively demonstrate efficacy of using this novel disinfection UV approach for treating lower concentrations of infectious oocysts under dynamic conditions found in WWTPs and in the aquatic environment. Development of a reliable and repeatable method of measuring fluence values from UV-delivered pulses for water disinfection applications is also required, and should take on board critical interrelated factors identified previously by Bolton and Linden [54] for continuous low- (LP) and medium-pressure (MP) UV units. 

Development of PUV has recently received attention as a potentially novel strategy for decontaminating water as it offers many benefits including rapid microbial reductions and efficiency of energy usage due to underpinning high peak-power dissipation during treatments [55, 56]. Indeed, use of ultraviolet (UV) light have become widely accepted as alternative methods to chlorination for wastewater disinfection [57, 58]. There are also over 2,000 wastewater treatment plants worldwide using either LP or MP ultra-violet technologies. Recent studies investigating continuous-use UV lamp technology has demonstrated the effectiveness of UV in inactivating pathogens in wastewater [57]. Research has also shown that, to ensure permanent inactivation and prevent the recovery of microorganisms following exposure to UV, a broad polychromatic spectrum of UV wavelengths is necessary such as doses delivered by MP and PUV systems. These wavelengths inflict irreparable damage not only on cellular DNA, but on other molecules such as enzymes as well. Moreover, numerous studies have also highlighted limitations of decontamination techniques such as conventional low pressure mercury lamps designed to produce energy at 254 nm (called monochromatic or germicidal light) that include microbial repair and the necessity for lengthy durations of exposure to obtain suitable levels of decontamination [59]. More recently, medium-pressure mercury UV lamps have been used because of their much higher germicidal UV power per unit length and because of their ability to emit polychromatic light comprising germicidal wavelengths from 200 to 300 nm) [54]. This approach kills microorganisms by using ultrashort duration pulses of an intense broadband emission spectrum that is rich in UV-C germicidal light (200 to 280 nm band). 

PUV is produced using techniques that multiply power manifold by storing electricity in a capacitor over relatively long times (fractions of a second) and releasing it in a short time (millionths or thousandths of a second) using sophisticated pulse compression techniques [56, 60, 61]. The emitted flash has a high peak power and usually consists of wavelengths from 200 to 1100 nm broad spectrum light enriched with shorter germicidal wavelengths [62, 63]. This technology has received several names in the scientific literature: pulsed UV light [64–66], high intensity broad-spectrum pulsed light [67], pulsed light [60], intense pulsed light [56], and pulsed white light [68]. Despite the fact that UV light appears effective for inactivating waterborne enteropathogens, including *Cryptosporidium* spp. it is recognised that many organisms have mechanisms for repairing light-induced DNA damage. However, Rochelle et al. [69] recently reported that *Cryptosporidium* spp. oocysts could neither repair UV light-induced damage nor regain infectivity under standard conditions used for storage and distribution of treated drinking water. This is despite the fact that both *C. parvum* and *C. hominis* were shown to harbour genes encoding UV repair proteins for mechanisms such as nucleotide excision repair and photolyase enzymes [70]. Indeed, irradiated oocysts were unable to regain preirradiation levels of infectivity, following exposure to broad array of potential repair conditions, such as prolonged incubation, preinfection excystation triggers, and post-UV holding periods. Otaki et al. [71] reported that adaptive microbial survival (tailing phenomenon) occurs when samples are treated in high turbidity solutions using continuous UV sources whereas tailing did not occur when similar samples were treated with pulsed xenon lamp. In terms of potential future alleviation strategies for combating *C. parvum* in water, it is likely the combined use MP and PUV technologies would offer considerable advantages as the next-generation decontamination bolt-on approach including rapid processing and efficiency of oocyst destruction, and should accommodate dynamic operational requirements at WWTP level. 

Use of ozone is gaining in popularity as an alternative or complementary approach for disinfection in drinking water facilities worldwide [58]. Some landmark studies have recently reported on the possible efficacy of using ozone for destroying *Cryptosporidium* oocysts [45]. However, there are very limited published findings to date that holistically investigate critical factors governing the effective and repeatable destruction of this recalcitrant enteroparasite in drinking water supplies using ozone or other oxidative agents. Of those researchers that have reported on this complex oxidation process, it would appear that using ozone singly or combined with (or without) free chlorine or monochloramine (in addition to other synergistic factors such as disinfection concentration, contact time, dissolved organic content concentration, pH, and temperature) are of critical importance for the inactivation of treated *C. parvum* [72, 73]. Despite limited understanding of the dynamic complexities underpinning advanced oxidation processes, ozonation is often preferred to chlorination because former leads to smaller concentrations of potentially harmful halogenated disinfection by-products. Yargeau and Leclair [74] also recently reported that use of ozone appears to be a promising technique for degradation of antibiotics, even in wastewater. After 4.5 min of ozone treatment, the concentration of sulphamethoxazole was below the HPLC detection limit of 0.6 mgL^−1^ indicating a degradation efficiency higher than 99.24%. Intense research has been recently focused on the development of novel pulsed plasma gas discharge technology as a complementary means of treating water containing unwanted microbial pathogens and chemicals. 

**Figure 1:**
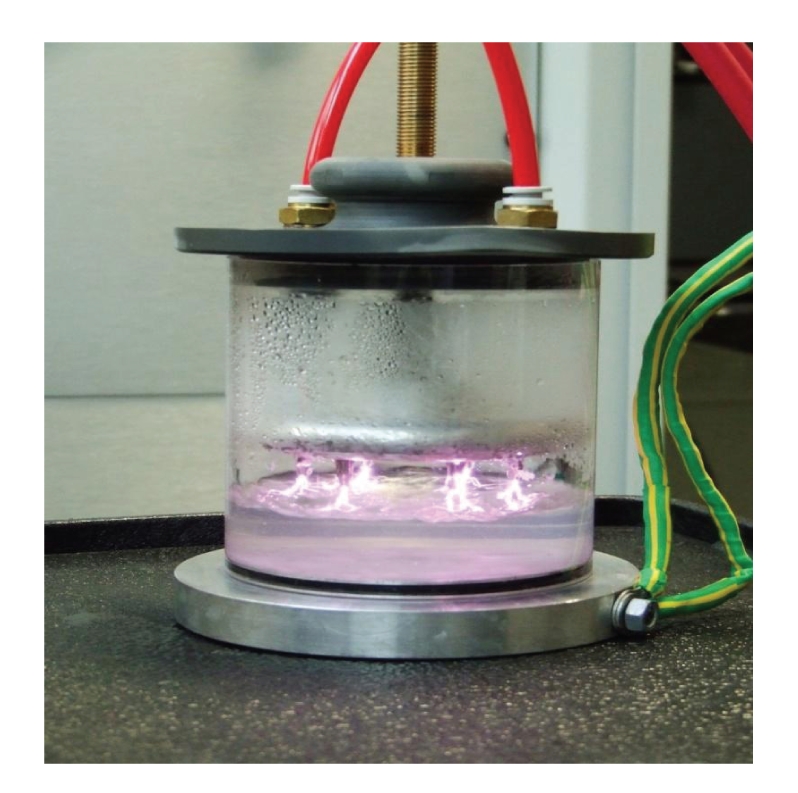
Pulsed-plasma gasdischarge decontamination treatment of water.

Pulsed plasma gas-discharge (PPGD) technology involves applying high voltage pulses to gas-injected test liquids resulting in the formation of a plasma that causes free radicals such as dissolved ozone and hydrogen peroxide, free electrons, ultraviolet light (UV), acoustic shock waves, and electric fields at levels between 10–50 kV/cm to be generated in the test liquids (Figure 1). Rowan and coworkers [75] has been shown that application of PPGD successfully reduces unwanted *Campylobacter* and *Salmonella* spp. in poultry wash water. PPGD (akin to PUV) is an enabling technology that requires transient generation of high voltage and high current that in turn results in the generation of large peak powers ranging from Megawatts to Terrawatts. Depending upon the application (PPGD or PUV), a pulse generator will deliver a large energy level on a single shot basis or alternatively will deliver a modest amount of energy (1–10 J) at a repetition rate from 10 to 10,000 pulses per second. Thus PPGD offers a radical new approach to energy delivery that involves the use of repetitive switching techniques to deliver stored energy in intense ultrashort bursts (85–100 nanoseconds). During each pulse, very high levels of peak power are generated (10–20 MW), and treatment is achieved using the required number of pulses, which is favourable in terms of requirements for new energy efficient technologies for the fast-approaching post peak oil era. Other research groups have also reported on the successful use of pulsed high voltage for decontaminating microbial populations [76] and phenol [77] in liquid solutions. Interestingly, research in our laboratory has shown that the myriad of biocidal properties generated by use of PPGD technology has also demonstrated promising results for the removal of prions on surgical material intimating potentially other healthcare applications for this decontamination approach [78].

### 2.3. Potential Hurdles for New Technologies

However, a nonoptimized pulsed plasma-gas discharge process may also lead to the formation of undesirable by-products such as brominated organic compounds and halogenates in treated water [79]. Brominated organic compounds are also considered potentially harmful and remain the subject of international study in order to elucidate mechanistic and kinetic information regarding their formation during ozonation so as to identify effective strategies for their reduction or elimination [80]. Considerable attention should be given to reducing or eliminating the occurrence of harmful by-products of ozone during this project via adjustments in sparged-gas composition, decreasing pH and complementary use of pulsed UV technology. Surprisingly, despite increased interest in the development of nonthermal advanced oxidative processes (such as corona plasma discharges, ozone combined with H_2_O_2_, low/medium pressure UV combined with H_2_O_2_, etc.), this author has been unable to source any published reports on possible toxicological issues associated with use of these new technologies for disinfection applications in healthcare, agri-food or the environment. On a related point, our research group has recently reported on the occurrence of VBNC state for food and water-borne microorganisms generated in liquid suspensions after separate exposure to PPGD and pulse electric field technologies [6, 7].

## 3. Antibiotics in the Aquatic Environments

Although antibiotics have been used for decades, only recently has an increasing number of studies highlighted the lack of understanding and knowledge about persistence of antibiotics in the aquatic environment and the potential environmental risks that this may pose [81]. Particularly as antibiotics are often poorly degraded or removed in conventional wastewater treatment plants (WWTPs) which causes formation of toxic degradation products that may impact negatively on the aquatic environment and public health [81]. Antibiotics in the broader sense are chemotherapeutic agents that inhibit or abolish the growth of microorganisms, and have been used extensively in human and veterinary medicine as well as aquaculture for the purpose of preventing or treating microbial infections. Wise [82] estimated antibiotic consumption worldwide to lie between 100,000 and 200,000 ton per annum. The reader is also directed to the recently published landmark reviews of Kümmerer [83, 84] for further detailed information on the possible input, occurrence, fate, and effects of antibiotics in the aquatic environment. 

The concentrations of antibiotics in municipal sewage and in sewage treatment plants are typically lower by a factor of 100 compared to hospital effluent [85]. While bacterial resistance to antibiotics have also been found in soil [86]. Some studies revealed that many different types of antibiotics are biodegradable under aerobic or anaerobic conditions, while other related research has reported that certain aquatic microbes can actually use these free active compounds as a sole carbon source. However, research has also shown that the concentrations and activity spectrum of compounds found in the environment does not correlate with the presence of resistant bacteria isolated from the same environments. Concern over the development of secondary resistance to commonly-used antibiotics (either through vertical and/or horizontal gene transfer among related and unrelated bacteria) in the environment with potential for adversely affecting aquatic and terrestrial organisms along with the potential for a cyclic unwanted return to humans again via consumption of drinking water has stimulated numerous research groups to investigate these important issues. Kümmerer [83] clearly articulated that the issue of acquired resistance was nearly always addressed in publications by describing the presence of antibiotics in the environment, with often mere speculation as to the possible relationship between low concentrations of antibiotics in water and emergence of resistance. In truth, the latter is a highly complex process that is as yet not fully understood [87]. However, the trend of accumulating and accelerating resistance to antibiotics is of concern as this is also juxtaposed by the marked reduction in mankinds' current arsenal of effective tools for combating this phenomenon. 

From a review of best published literature it would appear that possibly the most significant undefined risk in terms of antibiotics are microorganisms harbouring multiple antibiotic resistance genes such as vancomycin-resistant enterococci (VRE), methicillin-resistant *Staphylococcus aureus* (MRSA), and multiresistant pseudomonads that are living in close proximity to each other (such as in biofilms—sewage sludge flocs) as opposed to the free active compounds present at low concentrations in the aquatic environment. Indeed, Davison [88] provided evidence that antibiotic resistance is already present in natural environments and that it can be exchanged between bacteria for at least a decade. Therefore, under intense discussion is the possibility that nutrient-poor, oxygen-limited and cold aquatic environments exemplified by sewage sludge may be selecting for and acting as a reservoir for slowing growing resistant organisms. This environment provides for a higher biodiversity of microbial types and numbers affording a greater probability for accelerated exchange of antimicrobial drug resistance and virulence determinants in the presence of other neighbouring microbes deficient in such deleterious properties. Indeed, Ohlsen et al. [89] revealed that antibiotics even at sub-inhibitory concentrations can have an impact on cell function and can stimulate expression of quiescent virulence factors or potentiate transfer to an antibiotic resistant state. The latter is strongly aligned with findings from unrelated environmental stress studies which previously showed that many food and waterborne microbial species can sense and adapt to related and unrelated sub-lethal environmental stresses (such as osmotic stress or starvation) through complex quorum sensing and respond through an orchestrated controlled subsequence of preferential gene expression [90]. Thus, not alone may the latter confer microbial tolerance to the applied stress through a process of elevating activities in important genes that regulate key house-keeping functions such as osmotic stress response and protection vital proteins (chaperone), but such environmental tempering may also act as stimuli for up-regulating microbial virulence factor expression. The classic example being *L. monocytogenes*, where its' transcriptional activator PrfA up-regulates virulence factor expression in the presence of a known stress (e.g., body temperature), yet down-regulates transcription of pathogenic determinants in the presence of soil-related carbohydrates. Interestingly, Zupan and Raspor [91] recently reported on the development of an invasion-agar assay for determining in vitro invasiveness of *Saccharomyces cerevisiae*, and showed that this yeast augmented virulence when exposed to temperatures typical of human fever (37 to 39°C) yet exhibited strong repression effect on invasion in the presence of salts, anoxia and some preservatives. The aforementioned also highlights the extreme versatility of microbial species present in complex communities (such as in biofilms in WWTPs) to rapidly change and adapt when confronted with a sustained external selective pressure. 

Kümmerer [83] stated that there is a dearth of information on the interrelated factors governing the fate and effects of antibiotics in the environment (i.e., microbial ecology), highlighting that the majority of published studies to date are limited to single compound approaches. He also recently postulated that the effects of antibiotics in WWTPs or in the aquatic environment may be under-estimated, particularly as there is evidence that microbial exposure to antibiotics from the same group or from different groups may result in additive effects. For *β*-lactams it has been shown that their potency is much higher in the presence of 5-fluorouracil, a cytotoxic compound also present in sewage in concentrations at the mg or *μ*gL^−1^ range. Use of biocides such as triclosan and quaternary ammonium compounds used in hospitals and homes may also select for antibiotic resistance in microbial pathogens. Additionally, there is no published information as yet on the potential impact of low levels of other pharmaceutically active compounds (such as endocrine disrupting chemicals) on development of antibiotic resistance as the former also tolerate sewage treatment. 

There is also limited information on how long bacteria maintain antibiotic resistance in the absence of continued selective pressure for that resistance. That said, recent findings also suggest that bacteria which have already have become resistant through the application of antibiotics will not necessarily have a selective advantage in sewage treatment [92, 93]. Interestingly Kümmerer [83] reported that resistance was found to be high in hospital effluents and in sewage treatment plants, yet hospital effluents contribute to less than 1% of the total amount of municipal waste suggesting that hospitals are not the main source of resistant bacteria in municipal sewage. The latter would also suggest that antibiotic usage in the community accounts for the main input of resistant bacteria into municipal sewage. The question should also be posed as to whether or not multi-drug resistant bacteria that transcend to the quiescent VBNC state after periods of extended exposure to a nutrient depleted stressful environment (such as in water or in soil) are still capable of gene transfer and whether or not these important molecular determinants remain unaltered and stable. Kümmerer [84] also reported that the concentration of antibiotics may be much higher if the active compounds are persistent and accumulate, for example, by sorption to solid surfaces such sewage sludge, sediments, or soil. 

While most antibiotics tested to date have not been biodegradable under aerobic conditions(Kümmerer [83, 94, 95]),biodegradability has been poor for most of the compounds investigated in laboratory tests, including some to the *β*-lactams [96]. Out of 16 antibiotics tested, only benzyl penicillin (penicillin G) was completely mineralized in a combination test [97]. No evidence of biodegradation for tetracycline was observed during a biodegradability test, and sorption was found to be the principal removal mechanisms for tetracycline in activated sludge [98]. Substances extensively applied in fish farming had long half-lives in soil and sediment as reported in several investigations [99]. However, some substances were at least partly degradable [100]. Maki et al. [101] found that ampicillin, deoxycycline, oxytetracycline, and thiamphenicol were significantly degraded, while josamycin remained at initial levels. Despite the aforegoing, it has yet to be established that permanent exposure of antibiotics in sewage treatment systems promotes the development of antibiotic resistance and selective effects on bacterial communities. 

Studies have also revealed that bacteria that are resistant to antibiotics are present in surface water. Furthermore, antimicrobial resistance has also been found in marine bacteria [102] and bacteria living in estuaries or coastal waters polluted with sewage water [103]. Other researchers have reported that antibiotic resistance genes (ARGs) can be found in a region where no selection pressure exists [104]. Whereas Pruden et al. [105] noted that tetracycline ARGs tet(W) and tet(o) were present in treated drinking water and recycled wastewater, suggesting that these are potential pathways for the spread of ARGs to and from humans. High loads of antibiotics in sediments at concentrations potent enough to inhibit the growth of bacteria have been reported for aquaculture [83]. While antibiotic-resistant bacteria have been detected in drinking water supplies as far back as the 1980s. In fish farming sector such as aquaculture and mariculture, the widespread use of antibiotics for treating bacterial diseases has been associated with the development of resistance in a range of bacterial pathogens including *Aeromonas hydrophilia, Aeromonas salmonicida, Edwardsiella tarda, Edwardsiella icttaluri, Vibrio anguillarum, Vibrio salmonicida, Pasteurella piscida* and *Yersinia ruckeri* [106]. There are also considerable gaps in current knowledge concerning the possible transfer of chemical contaminants and microbial pathogens (including those harbouring ARGs) into the food chain through land spreading of some treated organic municipal and industrial material on agricultural land used for food production. The reader is directed to the comprehensive report produced recently by the Food Safety Authority of Ireland that addresses critical issues underpinning these putative concerns [107].

Other Pharmaceutically Active chemicals. A marked observation from this paper is the pressing need for greater risk-based assessment and for new and/or improved alleviation strategies for the optimal decontamination of wastewater at treatment plant level so as to reduce or eliminate the microbial (and their associated metabolite) risks to public health. In the context of improving wastewater treatment and planning for safe drinking supplies, one must also acknowledge growing international concerns about the release of certain chemicals into the aquatic environment that may result in alterations in the reproductive health of humans and wildlife [108–110]. The environmental presence of such man-made and naturally occurring compounds, properly referred to as endocrine disrupting chemicals (EDCs), can mimic or interfere with the binding and action of natural hormones, thus disrupting normal physiological processes [111, 112]. Consumption of water contaminated with EDCs may also cause reproductive disorders in humans as such chemicals have the ability to mimic the function of natural estrogens as well as disrupting the synthesis and metabolism of hormones by binding to hormonal receptors (cited in [108, 110]). Thyroid system-disrupting activity in water from municipal domestic sewage treatment plants was also detected recently [112].The list of known EDCs is extensive and includes natural and synthetic steroid hormones (such as 17 *β*-estradiol(E_2_),estrone (E_1_), and 17 *α*-ethinylestradiol (EE_2_)), phytoextrogens, pesticides, pharmaceuticals, and surfactants, all of which have been detected worldwide in processed water from domestic treatment plants (WWTPs) at the ng/L level that may cause abnormalities to aquatic organisms [113, 114]. EDCs were recently detected in effluents from WWTPs and from receiving waters at levels exceeding ng/L in Ireland [115]. 

Recent studies conducted by Fogarty and McGee [115] have demonstrated that fish habituating downstream of waste-water treatment plants (WWTPs) in the Midlands region in Ireland exhibited delayed spermatogenesis compared with fish upstream and intersex (feminization) was discovered in roach [115–117]. The scientific community has particularly focused on estrogenic EDCs (i.e., compounds interacting with the human estrogen receptor *α*), which enter the environment from a variety of sources including effluent discharge pipes, agricultural runoff and landfills [117]. In particular, such EDCs are considered to be dominant contributors to estrogenic activity in treated water from WWTPs and have been found in treated effluent at the ng/L level [113, 118]. Whilst intensive conventional-based treatment approaches have investigated many interrelated factors in the design and operating conditions of WWTPs for reduction or elimination of EDCs in treated wastewater, none have reported on their effective removal. Moreover, due to the common practice of residual chlorine in drinking water distribution, halongated by-products such as haloacetic acids and trihalomethanes form that exhibit a range of potential carcinogenic potentials (cited in [119]). The European commission (COM [1999] 706) acknowledged that there is an urgent need for further scientific research to denature deconjugated EDCs in the environment. Therefore, it is essential that such compounds be efficiently and effectively removed from processed water discharged from WWTPs. On a similar theme, Yamamoto and coworkers [120] recently reported the persistence of 8 pharmaceuticals with relatively high ecological risk and high consumption (namely acetaminophen, atenolol, carbamazepine, ibuprofen, ifenprodil, indomethacin,metenamicacid, and propranolol) using river water.

Previous researchers have recently shown that use of similar non-thermal corona discharge processes such as the PPGD technology may be potentially effective at destroying structurally-related organic compounds such as dyes, phenol and aniline in aqueous solutions [121–125]. Other nonplasma related studies have shown that the use of UVA can also facilitate removal of EDCs including E_1_, E_2_, and EE_2_ from water by photolysis [123, 126]. Given that the majority of known EDCs are phenolic in chemical structure, the benzene rings underpinning these EDCs should be rapidly photodegraded by hydroxyl radical attack during PPGD treatments. It is worth noting that the use of ozone become widely accepted as alternative methods to chlorination for wastewater disinfection [57, 58], and ozone has traditionally been applied in drinking water treatment plants for disinfection [58]. While other researchers have demonstrated the production of short-lived, high-oxidative, plasmochemical elements in pulsed-plasma-treated test liquids such as water and effluent for bacterial decontamination, no study to date has reported on the use of corona discharges singly or combined with pulsed UV light as a novel approach for the destruction of established and emerging microbial threats to public health in water or effluents. These dynamic environmental fate studies must be carried out in tandem with experiments that ascertain the likelihood of by-product toxicant formation and unwanted persistence in water, and address these associated undefined risks by either adjusting existing and/or implementing new complementary alleviation strategies.

## 4. Conclusion and Outlooks

There is a pressing need to generate critical data in gaps highlighted above via provision of funding to establish consortia of meaningful stakeholders comprising scientists, engineers, and end-users in order to facilitate policy makers and implementers (suchaswater managers) strive towards meeting ambitious objectives set for the EU's Water Framework Directive that requires member states to attain a good ecological status for all water bodies by 2015 [127]. While a global response to emerging environmental problems has been positive with the provision of substantial research funding been made available to the broad and diverse researcher communities (such as EU FP7 initiatives), one obvious real challenge that still remains is how does mankind harness and channel vast amounts of relevant sophisticated data that is produced and disseminated from a multiplicity of research groups worldwide into meaningful streamlined forums that can be shared and understood by all in a timely fashion. Exploiting advances in information technology will assist greatly with this challenge and will drive effective risk-based assessment, evaluation, and management of present and emerging problems for the aquatic environment. 

Currently, there is insufficient information available to reach definitive conclusion on the significance and impact of the VBNC state organisms and antibiotic resistant bacteria in the environment which would allow for the assessment of the potential risk related, for instance, to human health and ecosystem functions. Indeed impact of antibiotics present in the aquatic environment on the frequency of resistance transfer is questionable, with greater concern placed on the input of resistant bacteria into the environment from different sources. This suggests that greater emphasis should be placed on reducing the likelihood of antibiotic resistance occurrence in the first instance at the point of source by prudent management and careful rotation of antibiotic usage in both the hospital and community settings and by systematic continued monitoring of resistance, which will collectively impact positively on public health and wellbeing. The lack of understanding and knowledge surrounding a broad range of established and emerging risks to the aquatic environment is apparent. For example, Mena and Gerba [128] recently reviewed best published data for risk assessing the opportunist pathogen *P. aeruginosa* in water and concluded that process of estimating risk is currently significantly constrained because of the absence of specific (quantitative) occurrence data for Pseudomonads. 

In order to gain a greater appreciation and understanding of the critical environmental fate and associated impact of such undefined and variable microbial risks, more controlled laboratory investigations are needed to be undertaken in combination with conducting field studies. There is also pressing need to obtain definitive data on proposed risk to public health and to the aquatic environment combined with developing appropriate short- and long-term mathematical and computer models with capacity for monitoring and predicting ecotoxicological effects of these microbial stressors, particularly under dynamic naturalistic settings akin to those recently described by Bontje and coworkers [129] and Gevaert et al. [130] so as to inform policy makers and managers. It is quite apparent that proper judgement of the impact of microbial pathogens, their metabolites, and pharmaceutically active compounds require a thorough evaluation of their risk and hazard. Regarding the latter, where risk is normally expressed as the ratio between the predicted environmental concentration of the active ingredient (AI) and its predicted no-effect concentration. Hazard is expressed in terms of AI's persistence, potential for bioaccumulation, and ecotoxicity [131]. The combined use of monitoring data aligned with development and application of dynamic pollutant (contaminant) fate models is recommended. Pharmaceutical producers should also highlight environmental precaution when designing new more environmental friendly AIs, and that the environmental data should be transparent to the general public. In terms of holistic monitoring and prediction, we must also factor in seasonal environmental changes such as atmospheric and oceanic processes that occur in response to increasing greenhouse gases to map disease potentiation dynamics as this will aid development of appropriate strategies for controlling microbial risks across a range of human and natural systems. Indeed, Sedas [132] recently showed that climate also influences the abundance and ecology of pathogens, and the links between pathogens and changing ocean conditions, including human disease such as cholera. It is recognised that environmental risk-based management is typically uncertain due to different perceptions of the risk problem and our limited knowledge about the inter-play of biological, physical and chemical processes underlying these risks [133]. While a plethora of real-time quantitative microbial detection tools combined with more rapid and efficient decontamination approaches are on the horizon, we must be equally prudent about exhaustively confirming their efficacy such as agreed standards for sensitivity and reliability for detection, and eco-friendliness for decontamination. 

It is clear and apparent that the emergence of such complex problems has heralded a new dawn in research and innovation (i.e., a marked departure from the solitary PhD student environment), where collection, analysis and timely dissemination of such vast and meaningful information can only be effectively addressed using a well-managed consortia of networked researchers from various complementary disciplines by way of using a plethora of appropriate frontier funding initiatives such those offered by the EU (http://cordis.europa.eu/fp7/home.html). While it is recognised that undertaking such far-reaching cross-boundary initiatives will enable the potential impact, implications and future proofing of established and emerging risks to be managed and catered for properly. Is must be equally recognised that effectively managing and harnessing the potential of such diverse consortia comprising academics, industrial partners (SMEs to multinationals), policy makers and so forth pose significant logistic and complex challenges, for example, attempting to holistically cater for all stakeholders in a united global society on a single theme who have different needs and goals are real challenges. Therefore, such important issues must also be managed with a strong over-arching foresight, particularly in the context of embracing and exploiting advances in the communication and information technology landscape so that we can accommodate and provide for the real-time flow of knowledge to all stakeholders in order to identify potential synergies, emerging trends (problematic, beneficial or otherwise), and/or opportunities. 

## Figures and Tables

**Table 1:** Methods used to detect VBNC state in waterborne microorganisms.

Method(s) Employed	Reporting author(s)*
Failure of microbial growth in culture media	[5]
Use of redox probes to detect microbial respiratory chain activity	[6, 7]
Incorporation of radio-labelled substrates in culture media	[8]
Resuscitation in embryo of egg yolk	[4]
Detection in immunodeficient mice	[9]
Addition of antioxidants to culture media	[10]
RNA-based genotypic approaches (16S/23S rRNA, mRNA)	[11]
cDNA microarrays	[2]
*In situ* hybridisation (FISH), microradiography, epi-fluorescence microscopy, flow cytometry	[12]
Rapid enzyme assays	[13]
Oligonucleotide probes and tagged green fluorescent protein	[14]
Microbial quorum sensing	[15]

*This is a representative list of authors citing use of named methods for detection of VBNC state in waterborne organisms and therefore does not convey all published work in this area.

## References

[1] Byrd JJ, Xu H-S, Colwell RR (1991). Viable but nonculturable bacteria in drinking water. *Applied and Environmental Microbiology*.

[2] Dunaev T, Alanya S, Duran M (2008). Use of RNA-based genotypic approaches for quantification of viable but non-culturable Salmonella sp. in biosolids. *Water Science and Technology*.

[3] Sawaya K, Kaneko N, Fukushi K, Yaguchi J (2008). Behaviors of physiologically active bacteria in water environment and chlorine disinfection. *Water Science and Technology*.

[4] Cappelier JM, Besnard V, Roche SM, Velge P, Federighi M (2007). Avirulent viable but non culturable cells of *Listeria monocytogenes* need the presence of an embryo to be recovered in egg yolk and regain virulence after recovery. *Veterinary Research*.

[5] Xu HS, Robert N, Singleton FL, Attwel RW, Grimes DJ, Colwell RR (1982). Survival and viability of non-culturable *Escherichia coli* and *Vibrio cholera* in the estuarine and marine environment. *Microbial Ecology*.

[6] Yaqub S, Anderson JG, MacGregor SJ, Rowan NJ (2004). Use of a fluorescent viability stain to assess lethal and sublethal injury in food-borne bacteria exposed to high-intensity pulsed electric fields. *Letters in Applied Microbiology*.

[7] Rowan NJ, Espie S, Harrower J (2008). Evidence of lethal and sublethal injury in food-borne bacterial pathogens exposed to high-intensity pulsed-plasma gas discharges. *Letters in Applied Microbiology*.

[8] Rollins DM, Colwell RR (1986). Viable but nonculturable stage of *Campylobacter jejuni* and its role in survival in the natural aquatic environment. *Applied and Environmental Microbiology*.

[9] Lindbäck T, Rottenberg ME, Roche SM, Rørvik LM (2010). The ability to enter into an avirulent viable but non-culturable (VBNC) form is widespread among *Listeria monocytogenes* isolates from salmon, patients and environment. *Veterinary Research*.

[10] Reissbrodt R, Rienaecker I, Romanova JM (2002). Resuscitation of *Salmonella enterica* serovar typhimurium and enterohemorrhagic *Escherichia coli* from the viable but nonculturable state by heat-stable enterobacterial autoinducer. *Applied and Environmental Microbiology*.

[11] Garcia-Armisen T, Servais P (2004). Combining direct viable counts (DVC) and fluorescent *in situ* hybridization (FISH) to enumerate viable *Escherichia coli* in rivers and wastewaters. *Water Science and Technology*.

[12] Servais P, Prats J, Passerat J, Garcia-Armisen T (2009). Abundance of culturable versus viable *Escherichia coli* in freshwater. *Canadian Journal of Microbiology*.

[13] Fiksdal L, Tryland I (2008). Application of rapid enzyme assay techniques for monitoring of microbial water quality. *Current Opinion in Biotechnology*.

[14] Bjergbæk LA, Roslev P (2005). Formation of nonculturable *Escherichia coli* in drinking water. *Journal of Applied Microbiology*.

[15] Brackman G, Celen S, Baruah K (2009). AI-2 quorum-sensing inhibitors affect the starvation response and reduce virulence in several *Vibrio* species, most likely by interfering with LuxPQ. *Microbiology*.

[16] Oliver JD, Kjelleberg S (1993). Formation of viable but nonculturable cells. *Starvation in Bacteria*.

[17] Besnard V, Federighi M, Cappelier JM (2000). Evidence of viable but non-culturable state in *Listeria monocytogenes* by direct viable count and CTC-DAPI double staining. *Food Microbiology*.

[18] Roszak DB, Colwell RR (1987). Metabolic activity of bacterial cells enumerated by direct viable count. *Applied and Environmental Microbiology*.

[19] Reezal A, McNeil B, Anderson JG (1998). Effect of low-osmolality nutrient media on growth and culturability of *Campylobacter* species. *Applied and Environmental Microbiology*.

[20] Cappelier JM, Magras C, Jouve JL, Federighi M (1999). Recovery of viable but non-culturable *Campylobacter jejuni* cells in two animal models. *Food Microbiology*.

[21] Rowan NJ, Kirf D, Tomkins P (2009). Studies on the susceptibility of different culture morphotypes of *Listeria monocytogenes* to uptake and survival in human polymorphonuclear leukocytes. *FEMS Immunology and Medical Microbiology*.

[22] Moreno Y, Piqueres P, Alonso JL, Jiménez A, González A, Ferrús MA (2007). Survival and viability of *Helicobacter pylori* after inoculation into chlorinated drinking water. *Water Research*.

[23] Kastbjerg VG, Nielsen DS, Arneborg N, Gram L (2009). Response of *Listeria monocytogenes* to disinfection stress at the single-cell and population levels as monitored by intracellular pH measurements and viable-cell counts. *Applied and Environmental Microbiology*.

[24] Kitajima M, Tohya Y, Matsubara K, Haramoto E, Utagawa E, Katayama H (2010). Chlorine inactivation of human novovirus, marin novovirus and poliovirus in drinking water. *Letters in Applied Microbiology*.

[25] Dodd CER, Sharman RL, Bloomfield SF, Booth IR, Stewart GSAB (1997). Inimical processes: bacterial self-destruction and sub-lethal injury. *Trends in Food Science and Technology*.

[26] Aldsworth TG, Sharman RL, Dodd CER, Stewart GSAB (1998). A competitive microflora increases the resistance of *Salmonella typhimurium* to inimical processes: evidence for a suicide response. *Applied and Environmental Microbiology*.

[27] EU Directive 2006/7/EC of the European Parliament and of the Council of 15 February 2006 concerning the management of bathing water quality and repealing directive 76/160/EEC.

[28] Deretic V, Levine B (2009). Autophagy, immunity, and microbial adaptations. *Cell Host and Microbe*.

[29] Tryland I, Pommepuy M, Fiksdal L (1998). Effect of chlorination on *β*-D-galactosidase activity of sewage bacteria and *Escherichia coli*. *Journal of Applied Microbiology*.

[30] Farnleitner AH, Hocke L, Beiwl C, Kavka GG, Mach RL (2002). Hydrolysis of 4-methylumbelliferyl-*β*-D-glucuronide in differing sample fractions of river waters and its implication for the detection of fecal pollution. *Water Research*.

[31] George I, Crop P, Servais P (2001). Use of *β*-D-galactosidase and *β*-D-glucuronidase activities for quantitative detection of total and fecal coliforms in wastewater. *Canadian Journal of Microbiology*.

[32] Caruso G, Crisafi E, Mancuso M (2002). Development of an enzyme assay for rapid assessment of *Escherichia coli* in seawaters. *Journal of Applied Microbiology*.

[33] George I, Anzil A, Servais P (2004). Quantification of fecal coliform inputs to aquatic systems through soil leaching. *Water Research*.

[34] Zimmerman AM, Rebarchik DM, Flowers AR, Williams JL, Grimes DJ (2009). *Escherichia coli* detection using mTEC agar and fluorescent antibody direct viable counting on coastal recreational water samples. *Letters in Applied Microbiology*.

[35] Okabe S, Shimazu Y (2007). Persistence of host-specific *Bacteroides-Prevotella* 16S rRNA genetic markers in environmental waters: effects of temperature and salinity. *Applied Microbiology and Biotechnology*.

[36] Smeets PWMH, Dullemont YJ, Van Gelder PHAJM, Van Dijk JC, Medema GJ (2008). Improved methods for modelling drinking water treatment in quantitative microbial risk assessment; a case study of *Campylobacter* reduction by filtration and ozonation. *Journal of Water and Health*.

[37] Madetoja J, Nystedt S, Wiklund T (2003). Survival and virulence of *Flavobacterium psychrophilum* in water microcosms. *FEMS Microbiology Ecology*.

[38] Madetoja J, Wiklund T (2002). Detection of the fish pathogen *Flavobacterium psychrophilum* in water from fish farms. *Systematic and Applied Microbiology*.

[39] Marco-Noales E, Biosca EG, Amaro C (1999). Effects of salinity and temperature on long-term survival of the eel pathogen *Vibrio vulnificus* biotype 2 (serovar E). *Applied and Environmental Microbiology*.

[40] Madsen L, Dalsgaard I (2008). Water recirculation and good management: potential methods to avoid disease outbreaks with *Flavobacterium psychrophilum*. *Journal of Fish Diseases*.

[41] Kooi BW, Bontje D, Liebig M (2008). Model analysis of a simple aquatic ecosystems with sublethal toxic effects. *Mathematical Biosciences and Engineering*.

[42] Noble RT, Weisberg SB (2005). A review of technologies for rapid detection of bacteria in recreational waters. *Journal of Water and Health*.

[43] Hunter PR, Syed Q (2001). Community surveys of self-reported diarrhoea can dramatically overestimate the size of outbreaks of waterborne cryptosporidiosis. *Water Science and Technology*.

[44] Rochelle PA, Marshall MM, Mead JR (2002). Comparison of in vitro cell culture and a mouse assay for measuring infectivity of *Cryptosporidium parvum*. *Applied and Environmental Microbiology*.

[45] Corona-Vasquez B, Samuelson A, Rennecker JL, Mariñas BJ (2002). Inactivation of *Cryptosporidium* parvum oocysts with ozone and free chlorine. *Water Research*.

[46] Slifko TR, Smith HV, Rose JB (2000). Emerging parasite zoonoses associated with water and food. *International Journal for Parasitology*.

[47] DuPont HL, Chappell CL, Sterling CR, Okhuysen PC, Rose JB, Jakubowski W (1995). The infectivity of *Cryptosporidium parvum* in healthy volunteers. *New England Journal of Medicine*.

[48] Craun GF, Nwachuku N, Calderon RL, Craun MF (2002). Outbreaks in drinking-water systems, 1991–1998. *Journal of Environmental Health*.

[49] McCuin RM, Clancy JL (2003). Modifications to United States Environmental Protection Agency methods 1622 and 1623 for detection of Cryptosporidium oocysts and Giardia cysts in water. *Applied and Environmental Microbiology*.

[50] Directive 98/83/CE relative a la qualite des eaux destinees a la consommation humaine.

[51] Haas CN, Rose JB (1995). Developing an action level for *Cryptosporidium*. *Journal of the American Water Works Association*.

[52] Garvey M, Farrell H, Cormican M, Rowan N (2010). Investigations of the relationship between use of in vitro cell culture-quantitative PCR and a mouse-based bioassay for evaluating critical factors affecting the disinfection performance of pulsed UV light for treating *Cryptosporidium parvum* oocysts in saline. *Journal of Microbiological Methods*.

[53] Johnson AM, Linden K, Ciociola KM, De Leon R, Widmer G, Rochelle PA (2005). UV inactivation of *Cryptosporidium hominis* as measured in cell culture. *Applied and Environmental Microbiology*.

[54] Bolton JR, Linden KG (2003). Standardization of methods for fluence (UV Dose) determination in bench-scale UV experiments. *Journal of Environmental Engineering*.

[55] Elmnasser N, Guillou S, Leroi F, Orange N, Bakhrouf A, Federighi M (2007). Pulsed-light system as a novel food decontamination technology: a review. *Canadian Journal of Microbiology*.

[56] Gómez-López VM, Ragaert P, Debevere J, Devlieghere F (2007). Pulsed light for food decontamination: a review. *Trends in Food Science and Technology*.

[57] Kalisvaart BF (2004). Re-use of wastewater: preventing the recovery of pathogens by using medium-pressure UV lamp technology. *Water Science and Technology*.

[58] Meunier L, Canonica S, von Gunten U (2006). Implications of sequential use of UV and ozone for drinking water quality. *Water Research*.

[59] Bintsis T, Litopoulou-Tzanetaki E, Robinson RK (2000). Existing and potential applications of ultraviolet light in the food industry—a critical review. *Journal of the Science of Food and Agriculture*.

[60] Rowan NJ, MacGregor SJ, Anderson JG, Fouracre RA, McIlvaney L, Farish O (1999). Pulsed-light inactivation of food-related microorganisms. *Applied and Environmental Microbiology*.

[61] Farrell H, Garvey M, Rowan N (2009). Studies on the inactivation of medically important *Candida* species on agar surfaces using pulsed light. *FEMS Yeast Research*.

[62] Lee S-U, Joung M, Yang D-J (2008). Pulsed-UV light inactivation of *Cryptosporidium parvum*. *Parasitology Research*.

[63] MacGregor SJ, Rowan NJ, McIlvaney L, Anderson JG, Fouracre RA, Farish O (1998). Light inactivation of food-related pathogenic bacteria using a pulsed power source. *Letters in Applied Microbiology*.

[64] Anderson JG, Rowan NJ, MacGregor SJ, Fouracre RA, Farish O (2000). Inactivation of food-borne enteropathogenic bacteria and spoilage fungi using pulsed-light. *IEEE Transactions on Plasma Science*.

[65] Sharma RR, Demirci A (2003). Inactivation of *Escherichia coli* O157:H7 on inoculated alfalfa seeds with pulsed ultraviolet light and response surface modeling. *Journal of Food Science*.

[66] Wang T, MacGregor SJ, Anderson JG, Woolsey GA (2005). Pulsed ultra-violet inactivation spectrum of *Escherichia coli*. *Water Research*.

[67] Roberts P, Hope A (2003). Virus inactivation by high intensity broad spectrum pulsed light. *Journal of Virological Methods*.

[68] Marquenie D, Geeraerd AH, Lammertyn J (2003). Combinations of pulsed white light and UV-C or mild heat treatment to inactivate conidia of *Botrytis cinerea* and *Monilia fructigena*. *International Journal of Food Microbiology*.

[69] Rochelle PA, Fallar D, Marshall MM, Montelone BA, Upton SJ, Woods K (2004). Irreversible UV inactivation of *Cryptosporidium* spp. despite the presence of UV repair genes. *Journal of Eukaryotic Microbiology*.

[70] Rochelle PA, Upton SJ, Montelone BA, Woods K (2005). The response of *Cryptosporidium parvum* to UV light. *Trends in Parasitology*.

[71] Otaki M, Okuda A, Tajima K, Iwasaki T, Kinoshita S, Ohgaki S (2003). Inactivation differences of microorganisms by low pressure UV and pulsed xenon lamps. *Water Science and Technology*.

[72] Driedger A, Staub E, Pinkernell U, Mariñas B, Köster W, Gunten UV (2001). Inactivation of *Bacillus subtilis* spores and formation of bromate during ozonation. *Water Research*.

[73] Rennecker JL, Mariñas BJ, Owens JH, Rice EW (1999). Inactivation of *Cryptosporidium parvum* oocysts with ozone. *Water Research*.

[74] Yargeau V, Leclair C (2007). Potential of ozonation for the degradation of antibiotics in wastewater. *Water Science and Technology*.

[75] Rowan NJ, Espie S, Harrower J, Anderson JG, Marsili L, MacGregor SJ (2007). Pulsed-plasma gas-discharge inactivation of microbial pathogens in chilled poultry wash water. *Journal of Food Protection*.

[76] Anpilov AM, Barkhudarov EM, Christofi N (2002). Pulsed high voltage electric discharge disinfection of microbially contaminated liquids. *Letters in Applied Microbiology*.

[77] Hoeben WFLM, Van Veldhuizen EM, Rutgers WR, Cramers CAMG, Kroesen GMW (2000). The degradation of aqueous phenol solutions by pulsed positive corona discharges. *Plasma Sources Science and Technology*.

[78] Rowan NJ, Laffey JG, Rogers M Studies on the removal of prions from surgical instruments using novel pulsed plasma gas discharge system.

[79] Von Gunten U (2003). Ozonation of drinking water—part II: disinfection and by-product formation in presence of bromide, iodide or chlorine. *Water Research*.

[80] Hammes F, Salhi E, Köster O, Kaiser H-P, Egli T, von Gunten U (2006). Mechanistic and kinetic evaluation of organic disinfection by-product and assimilable organic carbon (AOC) formation during the ozonation of drinking water. *Water Research*.

[81] De Bel E, Dewulf J, Witte BD, Van Langenhove H, Janssen C (2009). Influence of pH on the sonolysis of ciprofloxacin: biodegradability, ecotoxicity and antibiotic activity of its degradation products. *Chemosphere*.

[82] Wise R (2002). Antimicrobial resistance: priorities for action. *Journal of Antimicrobial Chemotherapy*.

[83] Kümmerer K (2009). Antibiotics in the aquatic environment—a review—part I. *Chemosphere*.

[84] Kümmerer K (2009). Antibiotics in the aquatic environment—a review—part II. *Chemosphere*.

[85] Caplin JL, Hanlon GW, Taylor HD (2008). Presence of vancomycin and ampicillin-resistant *Enterococcus faecium* of epidemic clonal complex-17 in wastewaters from the south coast of England. *Environmental Microbiology*.

[86] Schmidt H, Römbke J, Kümmerer K (2008). The ecotoxicological effects of pharmaceuticals (antibiotics and antiparasiticides) in the terrestrial environment—a review. *Pharmaceuticals in the Environment. Sources, Fate, Effects and Risks*.

[87] Alanis AJ (2005). Resistance to antibiotics: are we in the post-antibiotic era?. *Archives of Medical Research*.

[88] Davison J (1999). Genetic exchange between bacteria in the environment. *Plasmid*.

[89] Ohlsen K, Ternes T, Werner G (2003). Impact of antibiotics on conjugational resistance gene transfer in *Staphylococcus aureus* in sewage. *Environmental Microbiology*.

[90] Foster JW, Spector MP (1995). How *Salmonella* survive against the odds. *Annual Review of Microbiology*.

[91] Zupan J, Raspor P (2010). Invasive growth of *Saccharomyces cerevisiae* depends on environmental triggers: a quantitative model. *Yeast*.

[92] Wiethan J, Al-Ahmad A, Henninger A, Kümmerer K (2001). Simulation of the selection pressure of the antibiotics ciprofloxacin and ceftazidim in surface water with classical methods. *Vom Wasser*.

[93] Al-Ahmad A, Haiß A, Unger J, Brunswick-Tietze A, Wiethan J, Kümmerer K (2009). Effects of a realistic mixture of antibiotics on resistant and nonresistant sewage sludge bacteria in laboratory-scale treatment plants. *Archives of Environmental Contamination and Toxicology*.

[94] Gartiser S, Urich E, Alexy R, Kümmerer K (2007). Ultimate biodegradation and elimination of antibiotics in inherent tests. *Chemosphere*.

[95] Li D, Yang M, Hu J, Ren L, Zhang Y, Li K (2008). Determination and fate of oxytetracycline and related compounds in oxytetracycline production wastewater and the receiving river. *Environmental Toxicology and Chemistry*.

[96] Alexy R, Kümpel T, Kümmerer K (2004). Assessment of degradation of 18 antibiotics in the closed bottle test. *Chemosphere*.

[97] Gartiser S, Urich E, Alexy R, Kümmerer K (2007). Anaerobic inhibition and biodegradation of antibiotics in ISO test schemes. *Chemosphere*.

[98] Kim S, Eichhorn P, Jensen JN, Weber AS, Aga DS (2005). Removal of antibiotics in wastewater: effect of hydraulic and solid retention times on the fate of tetracycline in the activated sludge process. *Environmental Science and Technology*.

[99] Lai H-T, Chien Y-H, Lin J-S (2008). Long-term transformation of oxolinic acid in water from an eel pond. *Aquaculture*.

[100] Thiele-Bruhn S (2003). Pharmaceutical antibiotic compounds in soils—a review. *Journal of Plant Nutrition and Soil Science*.

[101] Maki T, Hasegawa H, Kitami H, Fumoto K, Munekage Y, Ueda K (2006). Bacterial degradation of antibiotic residues in marine fish farm sediments of Uranouchi Bay and phylogenetic analysis of antibiotic-degrading bacteria using 16S rDNA sequences. *Fisheries Science*.

[102] Neela FA, Nonaka L, Suzuki S (2007). The diversity of multi-drug resistance profiles in tetracycline-resistant *Vibrio* species isolated from coastal sediments and seawater. *Journal of Microbiology*.

[103] Kimiran-Erdem A, Arslan EO, Sanli Yurudu NO, Zeybek Z, Dogruoz N, Cotuk A (2007). Isolation and identification of Enterococci from seawater samples: assessment of their resistance to antibiotics and heavy metals. *Environmental Monitoring and Assessment*.

[104] Sjölund M, Bonnedahl J, Hernandez J (2008). Dissemination of multidrug-resistant bacteria into the Arctic. *Emerging Infectious Diseases*.

[105] Pruden A, Pei R, Storteboom H, Carlson KH (2006). Antibiotic resistance genes as emerging contaminants: studies in northern Colorado. *Environmental Science and Technology*.

[106] Serrano PH (2005). Responsible use of antibiotics in aquaculture. *Fisheries Technical Paper*.

[107] Food Safety Authority of Ireland Report on food safety implications of land spreading agriculture, municipal and industrial organic matter on agricultural land used for food production in Ireland. www.fsai.ie/WorkArea/DownloadAsset.aspx?id=8226.

[108] Sharpe RM (2001). Hormones and testis development and the possible adverse effects of environmental chemicals. *Toxicology Letters*.

[109] EPA (2005). *Final Report: Endocrine Disruptors in the Irish Aquatic Environment*.

[110] Fernandez MP, Campbell PM, Ikonomou MG, Devlin RH (2007). Assessment of environmental estrogens and the intersex/sex reversal capacity for Chinook salmon (Oncorhynchus tshawytscha) in primary and final municipal wastewater effluents. *Environment International*.

[111] Sharpe RM, Skakkebaek NE (1993). Are oestrogens involved in falling sperm counts and disorders of the male reproductive tract?. *Lancet*.

[112] Murata T, Yamauchi K (2008). 3,3′,5-triiodo-l-thyronine-like activity in effluents from domestic sewage treatment plants detected by in vitro and in vivo bioassays. *Toxicology and Applied Pharmacology*.

[113] Ternes TA, Stumpf M, Mueller J, Haberer K, Wilken R-D, Servos M (1999). Behavior and occurrence of estrogens in municipal sewage treatment plants—I. Investigations in Germany, Canada and Brazil. *Science of the Total Environment*.

[114] Lishman L, Smyth SA, Sarafin K (2006). Occurrence and reductions of pharmaceuticals and personal care products and estrogens by municipal wastewater treatment plants in Ontario, Canada. *Science of the Total Environment*.

[115] Fogarty A, McGee C (2007). Male or female: that is the question?. *The Irish Scientist*.

[116] Brennan SJ (2005). *The ecotoxicological assessment and microbial transformation of selected oestrogen mimics*.

[117] Brennan SJ, Brougham CA, Roche JJ, Fogarty AM (2006). Multi-generational effects of four selected environmental oestrogens on *Daphnia magna*. *Chemosphere*.

[118] Protoxkit F (1998). *Freshwater Toxicity Test with a Ciliate Protozoan. Standard Operating Procedure*.

[119] Reid AM, Brougham CA, Fogarty AM, Roche JJ (2007). An investigation into possible sources of phthalate contamination in the environmental analytical laboratory. *International Journal of Environmental Analytical Chemistry*.

[120] Yamamoto H, Nakamura Y, Moriguchi S (2009). Persistence and partitioning of eight selected pharmaceuticals in the aquatic environment: laboratory photolysis, biodegradation, and sorption experiments. *Water Research*.

[121] Willberg DM, Lang PS, Höchemer RH, Kratel A, Hoffmann MR (1996). Degradation of 4-chlorophenol, 3,4-dichloroaniline, and 2,4,6- trinitrotoluene in an electrohydraulic discharge reactor. *Environmental Science and Technology*.

[122] Bubnov AG, Grinevich VI, Kuvykin NA (2004). Phenol degradation features in aqueous solutions upon dielectric-barrier discharge treatment. *High Energy Chemistry*.

[123] Liu YJ, Jiang XZ (2005). Phenol degradation by a nonpulsed diaphragm glow discharge in an aqueous solution. *Environmental Science and Technology*.

[124] Hao X, Zhou M, Xin Q, Lei L (2007). Pulsed discharge plasma induced Fenton-like reactions for the enhancement of the degradation of 4-chlorophenol in water. *Chemosphere*.

[125] Amin MT, Ryu S, Park H (2007). Degradation of phenol and Bisphenol-A using discharged water generating system. *Journal of Water Supply: Research and Technology—AQUA*.

[126] Coleman HM, Routledge EJ, Sumpter JP, Eggins BR, Byrne JA (2004). Rapid loss of estrogenicity of steroid estrogens by UVA photolysis and photocatalysis over an immobilized titanium dioxide catalyst. *Water Research*.

[127] Vaes G, Willems P, Swartenbroekx P, Kramer K, de Lange W, Kober K (2009). Scientific-policy interfacing in support of the water framework directive implementation. *Water Science and Technology*.

[128] Mena KD, Gerba CP (2009). Risk assessment of *Pseudomonas aeruginosa* in water. *Reviews of Environmental Contamination and Toxicology*.

[129] Bontje D, Kooi BW, Liebig M, Kooijman SALM (2009). Modelling long-term ecotoxicological effects on an algal population under dynamic nutrient stress. *Water Research*.

[130] Gevaert V, Verdonck F, Benedetti L, De Keyser W, De Baets B (2009). Evaluating the usefulness of dynamic pollutant fate models for implementing the EU Water Framework Directive. *Chemosphere*.

[131] Wennmalm A, Gunnarsson B (2009). Pharmaceutical management through environmental product labelling in Sweden. *Environment International*.

[132] Sedas VT (2007). Influence of environmental factors on the presence of *Vibrio cholerae* in the marine environment: a climate link. *Journal of Infection in Developing Countries*.

[133] Ragas AMJ, Huijbregts MAJ, Henning-De Jong I, Leuven RS (2009). Uncertainty in environmental risk assessment: implications for risk-based management of river basins. *Integrated Environmental Assessment and Management*.

